# Refining orthognathic outcomes: 3D accuracy with patient-specific implants for facial asymmetry correction

**DOI:** 10.1016/j.jpra.2025.05.007

**Published:** 2025-05-16

**Authors:** Samanta Buchholzer, Florent Moissenet, Romain Aymon, Paolo Scolozzi

**Affiliations:** a Resident, Division of Oral and Maxillofacial Surgery, Department of Surgery, Faculty of Medicine, University of Geneva & University Hospitals of Geneva, Geneva, Switzerland; b Research assistant in biomechanics Kinesiology and Biomechanics Laboratories, Department of Surgery, University of Geneva & Geneva University Hospitals, Geneva, Switzerland; c Statistician, Division of Oral and Maxillofacial Surgery, Department of Surgery, Faculty of Medicine, University of Geneva & University Hospitals of Geneva, Geneva, Switzerland; d Head, Division of Oral and Maxillofacial Surgery, Department of Surgery, Faculty of Medicine, University of Geneva & University Hospitals of Geneva, Geneva, Switzerland

**Keywords:** Facial asymmetry, Patient-specific implants, Orthognathic surgery, CAD-CAM technology

## Abstract

This study evaluated the three-dimensional accuracy of maxillary and chin repositioning using patient-specific implants (PSIs) in non-syndromic patients with facial asymmetry. A retrospective cohort study was conducted at the University Hospital of Geneva between 2018 and 2023. Three-dimensional pre-operative planning and post-operative CT or CBCT scans of 21 patients with facial asymmetry were analyzed. Using *3D Slicer*, images were imported and segmented, and the anterior surfaces of the maxilla and chin were extracted via *Meshmixer. CloudCompare* software calculated the distance between post-operative and pre-operative surface models using one million sample points. Boxplots visualized the distribution of signed Euclidean distances, and accuracy was assessed based on the proportion of values within ±2 mm. The mean difference between planned and actual post-operative surfaces was -0.9 mm for the maxilla and -0.5 mm for the chin. The proportion of values within the accuracy threshold was for the maxilla >90 % in 13 patients (62 %), 80–90 % in 5 patients (23.8 %), 50–80 % in 2 patients (9.5 %), and <50 % in 1 patient (4.8 %). For the chin, >90 % accuracy was observed in 7 patients (58.4 %), 50–80 % in 4 patients (33.3 %), and <50 % in 1 patient (8.3 %). These findings suggest that PSIs provide accurate maxillary and chin repositioning, with higher precision in maxillary repositioning. This study highlights the potential of PSIs to improve surgical outcomes in orthognathic procedures for facial asymmetry cases.

## Introduction

A thorough understanding of facial asymmetry is essential for accurate diagnosis, treatment planning, and long-term stability. It is typically classified into three categories: 1) congenital, originating prenatally; 2) developmental, emerging during growth with often indeterminate causes; and 3) acquired, resulting from trauma, functional mandibular shifts, or pathological conditions such as unilateral condylar hyperplasia, and temporomandibular joint (TMJ) ankylosis.^1^, ^2^, ^3^, ^4^

Planning and executing maxillomandibular osteotomies is complex and technically demanding. Precise control of pitch, roll, and yaw movements is critical to achieving skeletal symmetry.^5^, ^6^, ^7^, ^8^ Inadequate management of these rotations may lead to residual asymmetry, impaired facial harmony, and suboptimal functional and aesthetic outcomes.^5^, ^6^, ^7^, ^8^, ^9^, ^10^

Conventional manual workflows often lead to cumulative planning inaccuracies. In contrast, recent advances such as virtual surgical planning (VSP), computer-aided design and manufacturing (CAD-CAM), and patient-specific implants (PSIs), have greatly enhanced surgical precision and reduced errors associated with traditional orthognathic surgery techniques.^8^, ^9^, ^10^

Among these innovations, three-dimensional (3D) printed splints have improved accuracy but still have limitations, primarily due to their inherent reliance on mandibular condylar repositioning within the glenoid fossa for maxillary fixation, which can introduce errors.^8^, ^9^, ^10^, ^11^

To address these challenges, PSIs were introduced in 2015 as a *waferless* or *splintless* alternative that provides greater autonomy in maxillary repositioning by eliminating the dependence on condylar positioning within the glenoid fossa.^10^, ^11^, ^12^, ^13^

Studies have consistently demonstrated strong agreement between preoperative planning and postoperative outcomes when using PSIs, with positive results reported in both early and more recent investigations.^11^, ^12^, ^13^, ^14^, ^15^, ^16^, ^17^, ^18^, ^19^ Despite growing use in orthognathic surgery, patient-specific implants (PSIs) have been mostly applied to non-asymmetrical deformities. Their role in correcting facial asymmetry remains underexplored, with limited focused studies. Existing literature often lacks specificity or centers on syndromic cases, leaving a significant gap in evidence regarding PSI accuracy, effectiveness, and limitations in non-syndromic facial asymmetry correction.^20^, ^21^, ^22^, ^23^, ^24^, ^25^, ^26^, ^27^^,^^34^^,^^35^ The knowledge gap is even more pronounced in the context of PSI genioplasty, where only a few studies have been reported, all of them limited to non-asymmetric cases.^27^, ^28^, ^29^

The purpose of this study was thus to evaluate the 3D accuracy of maxillary and chin repositioning using PSIs in patients with facial asymmetry. The authors hypothesized that PSIs would enable accurate, predictable outcomes closely aligning with pre-operative planning.

## Materials and methods

### Study design and sample

To address the research question, the authors designed and implemented a retrospective cohort study. The study adhered to the Strengthening the Reporting of Observational Studies in Epidemiology (STROBE) guidelines and was conducted in accordance with the Declaration of Helsinki and was approved by our local Ethical Board (Project ID 2018–00,754).

The study population was selected from a database of orthognathic patients at the University Hospital of Geneva, Switzerland between 2018 and 2023. The inclusion criteria were: 1) subjects older than 16 years, 2) with a non-syndromic facial asymmetry, 3) who underwent a bimaxillary orthognathic surgery with PSI for waferless maxillary repositioning following Le Fort I osteotomy, with or without PSI for chin repositioning following genioplasty, and 4) with a 1-year clinic-radiological follow-up (CBCT: cone beam CT scan). The exclusion criteria were: 1) subjects with syndromic craniofacial deformities and 2) with previous history of facial surgery and/or trauma.

Facial asymmetry was defined as a clinically and radiographically apparent deviation from facial midline symmetry, involving the chin, mandibular body, or maxillary structures, and confirmed through three-dimensional imaging and clinical examination.

The CAD/CAM PSI plates as well as cutting and drilling guides were planned according to the protocol detailed below.

### 3D virtual surgical planning

1) Image transfer and processing

CT or CBCT images in DICOM (Digital Imaging and Communications in Medicine) format were transferred and processed using dedicated software (PRO PLAN CMF™ ONLINE https://www.materialise.com/fr/medical/proplan-cmf). These images were segmented to obtain a 3D digital model of the maxillofacial skeleton. Occlusion was recorded directly from the 3D dental scan images or, alternatively, from the conventional plaster dental casts digitized using a high-resolution 3D optical scanner.

2) Three-dimensional virtual planning of maxillary positioning (waferless or splintless approach)

A virtual Le Fort I osteotomy was performed, aligning the upper mid-incisal line with the skeletal midline defined by a vertical plane passing through the nasion and anterior nasal spine. Yaw, roll, and pitch discrepancies were corrected, and the maxilla was virtually repositioned to achieve the planned symmetry and occlusion.

3) Three-dimensional virtual planning of mandibular positioning

The osteotomized 3D bony segments of the mandible were adjusted according to the new maxillary position to achieve an optimal dental intercuspidation.

4) Three-dimensional virtual planning of genioplasty

In cases where genioplasty was required to achieve the final facial symmetry, chin osteotomies were performed according to the planned movements, ensuring a safety margin from the mental nerves and the apices of the teeth.

### PSI 3D plates and surgical guides planning

A patient-specific digital 3D plate, along with a surgical cutting and drilling guide, was designed to replicate the planned maxillary movement. Similarly, patient-specific digital 3D plates and guides were designed for precise chin repositioning.

It is important to highlight that the decision was made not to use PSI plates for mandibular repositioning. This choice was driven by the inherent challenges of accurately replicating the virtually planned centric relation of the condyles during surgery. Such challenges could result in discrepancies between the planned and actual post-operative occlusion, which would not be amenable to the intra-operative correction. Consequently, our center currently chose to continue using a traditional acrylic splint to secure the final bite and “freehand” reposition the condyles within the fossa.

### Surgical procedure

#### Anesthesia and preparation

The surgical procedure was performed under nasoendotracheal general anesthesia. Patients were administered parenteral antibiotics, receiving either 1 g of amoxicillin/clavulanic acid or 600 mg of clindamycin in cases of penicillin allergy.

#### Le fort I osteotomy

**Figure 1:**
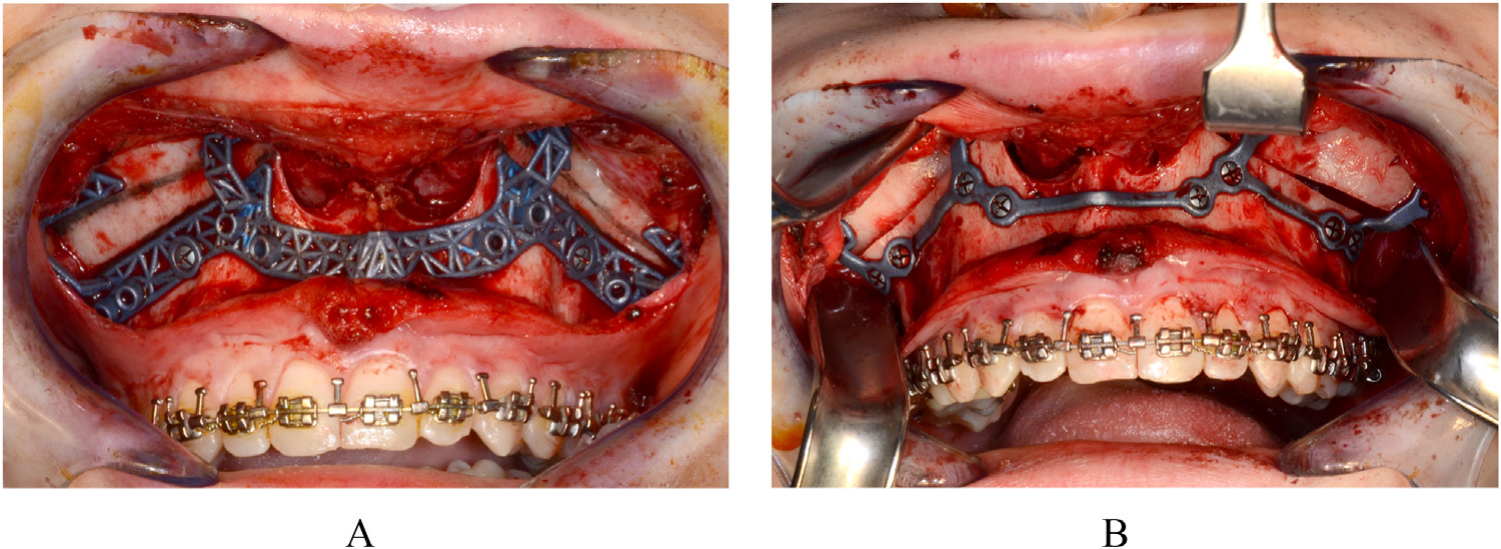
Intraoperative view of guided Le Fort I osteotomy. A) A cutting and drilling guide temporarily secured to the maxillary bone with monocortical screws. B) Maxillary repositioning and fixation using patient-specific 3D-printed plates.

The Le Fort I osteotomy was initiated by temporarily securing a cutting and drilling guide to the bone with monocortical screws. Maxillary osteotomies were then performed following the trajectory outlined by the guide (Figure 1a). Upon completion of the osteotomies, the guide was removed, and the maxilla was mobilized and repositioned according to the virtual surgical planning using patient-specific 3D plates for fixation (Figure 1b).

### Bilateral sagittal split osteotomy (BSSO)

The BSSO was performed utilizing the Epker technique.^30^ Bony interferences were removed to prevent mandibular segment collision, targeting the lingual cortex of the distal segment (short side) and buccal cortex of the proximal segment (long side, rotation side). The mandible and maxilla were secured in maxillomandibular fixation with steel wires positioned on a traditional acrylic splint. Osteosynthesis was achieved with two bicortical positioning screws.

### Genioplasty

**Figure 2:**
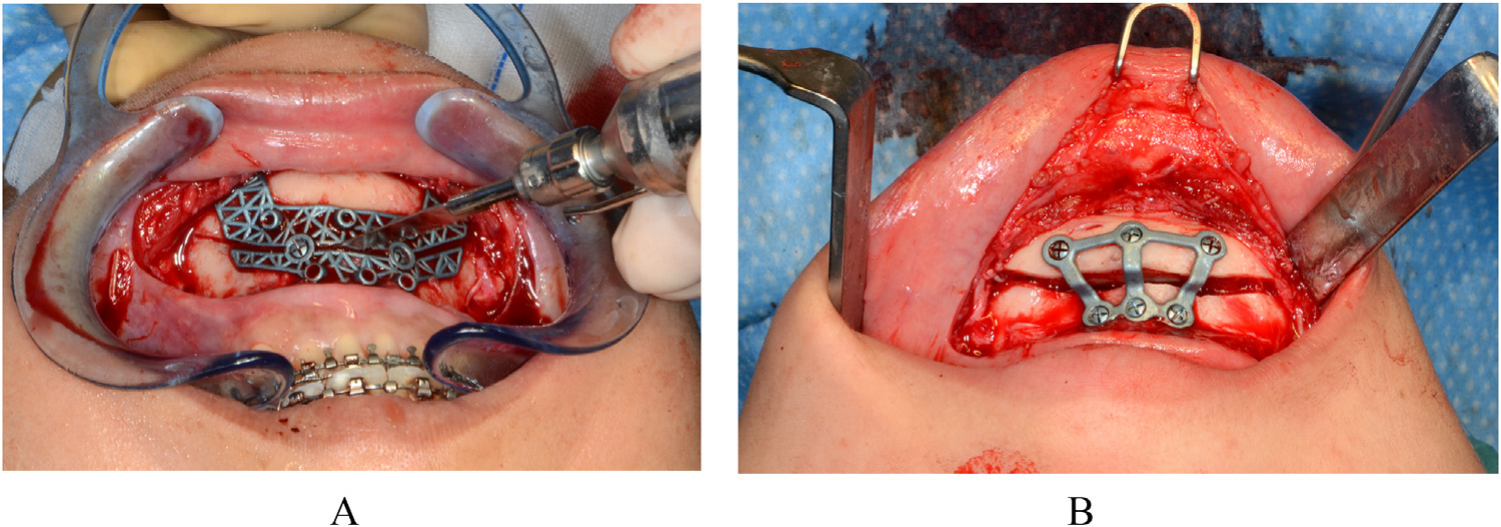
Intraoperative view of guided genioplasty. A) A cutting and drilling guide temporarily secured to the mandibular bone with monocortical screws. B) Chin repositioning and fixation using patient-specific 3D-printed plates.

For the genioplasty, a cutting and drilling guide was temporarily fixed to the bone using monocortical screws (Figure 2a). Chin osteotomies were performed along the trajectory defined by the guide. Following the completion of the osteotomies, the guide was removed, and the chin was mobilized and repositioned in alignment with the virtual surgical planning using patient-specific 3D plates for stabilization (Figure 2b).

### Post-operative accuracy assessment

**Figure 3:**
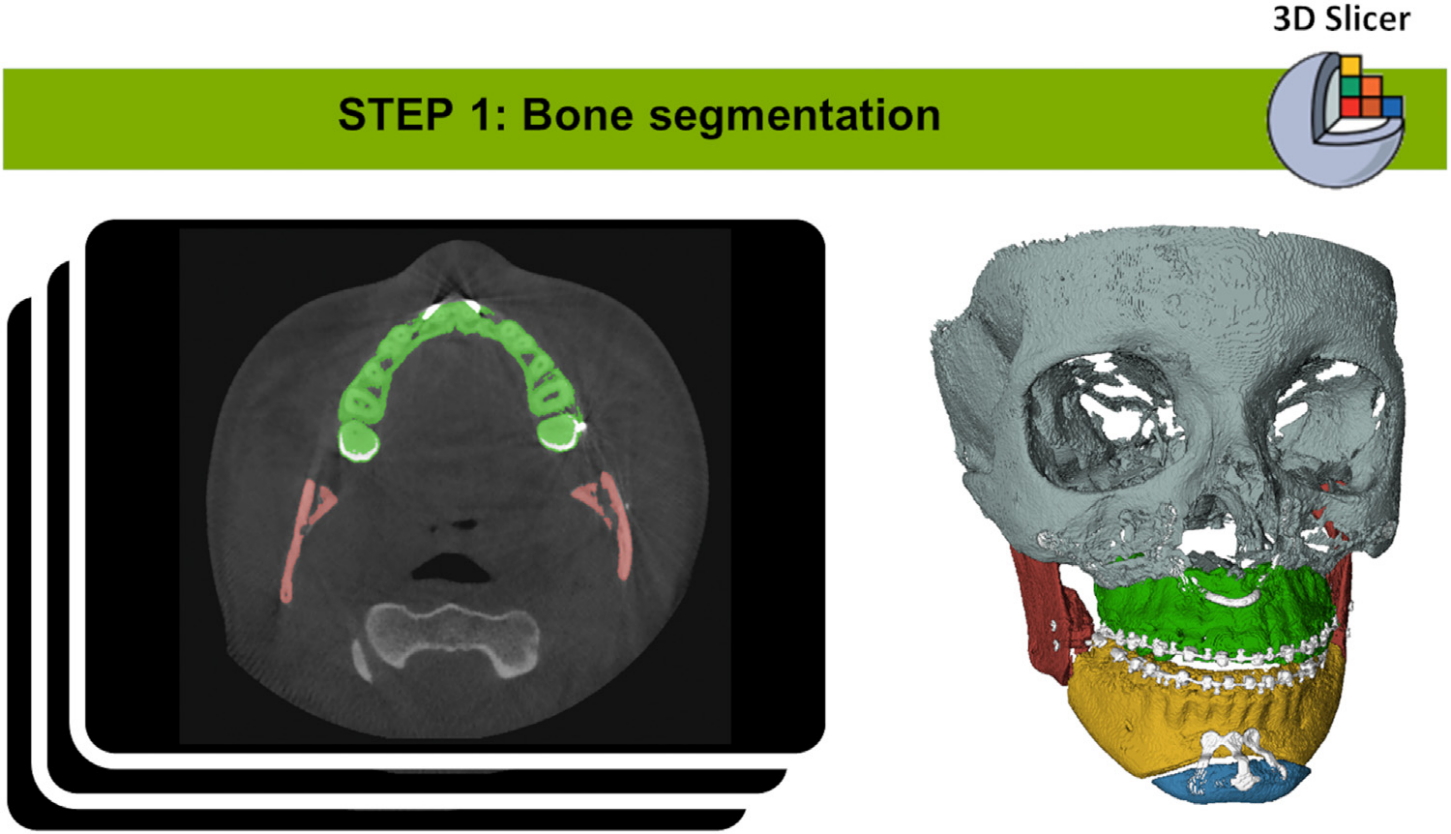
Bone segmentation of post-operative CT Images by using the *Segment Editor* module of 3D Slicer software.

**Figure 4:**
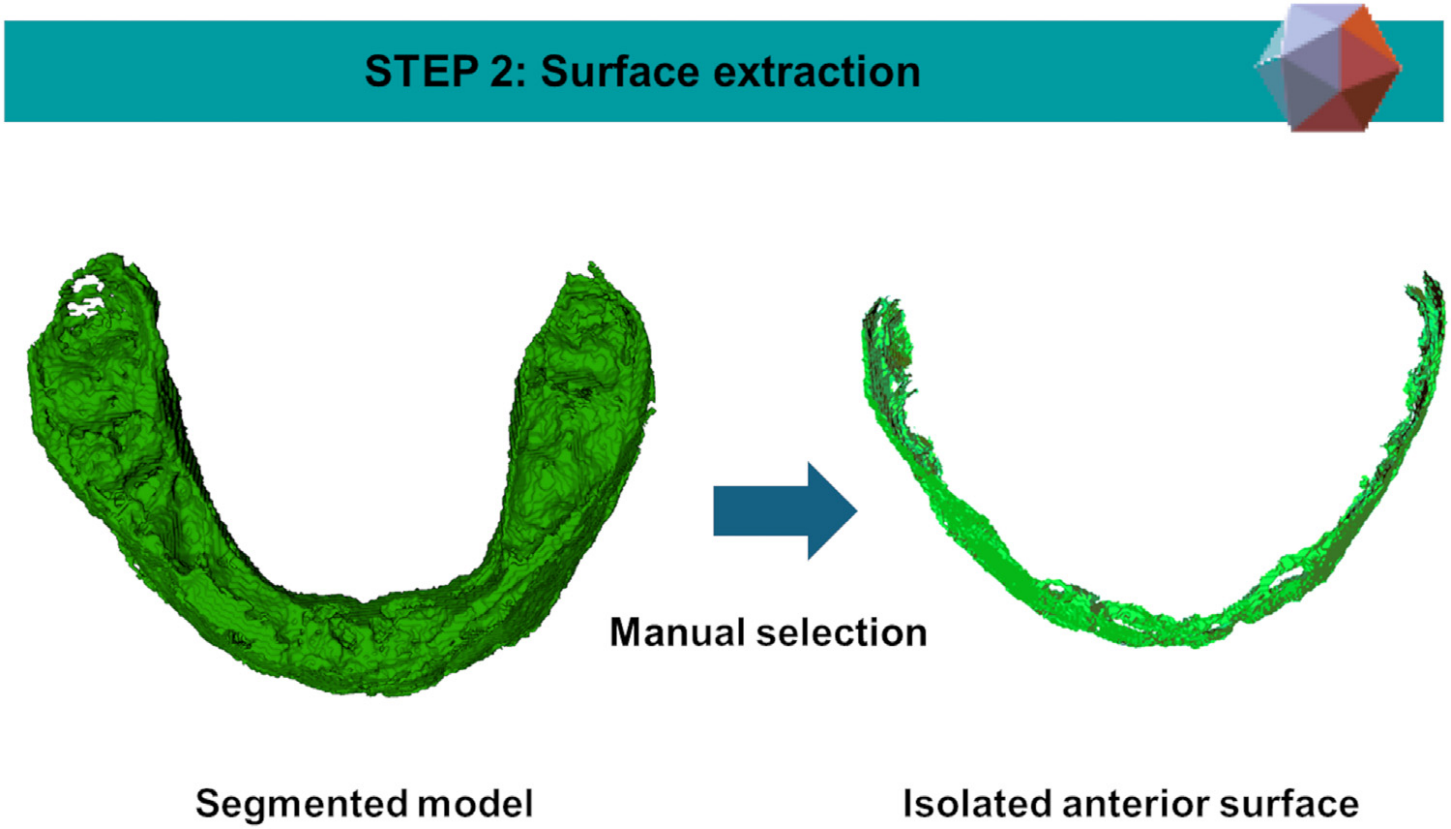
Extraction of the anterior surface of the previously segmented maxilla by using *Meshmixer* software.

**Figure 5:**
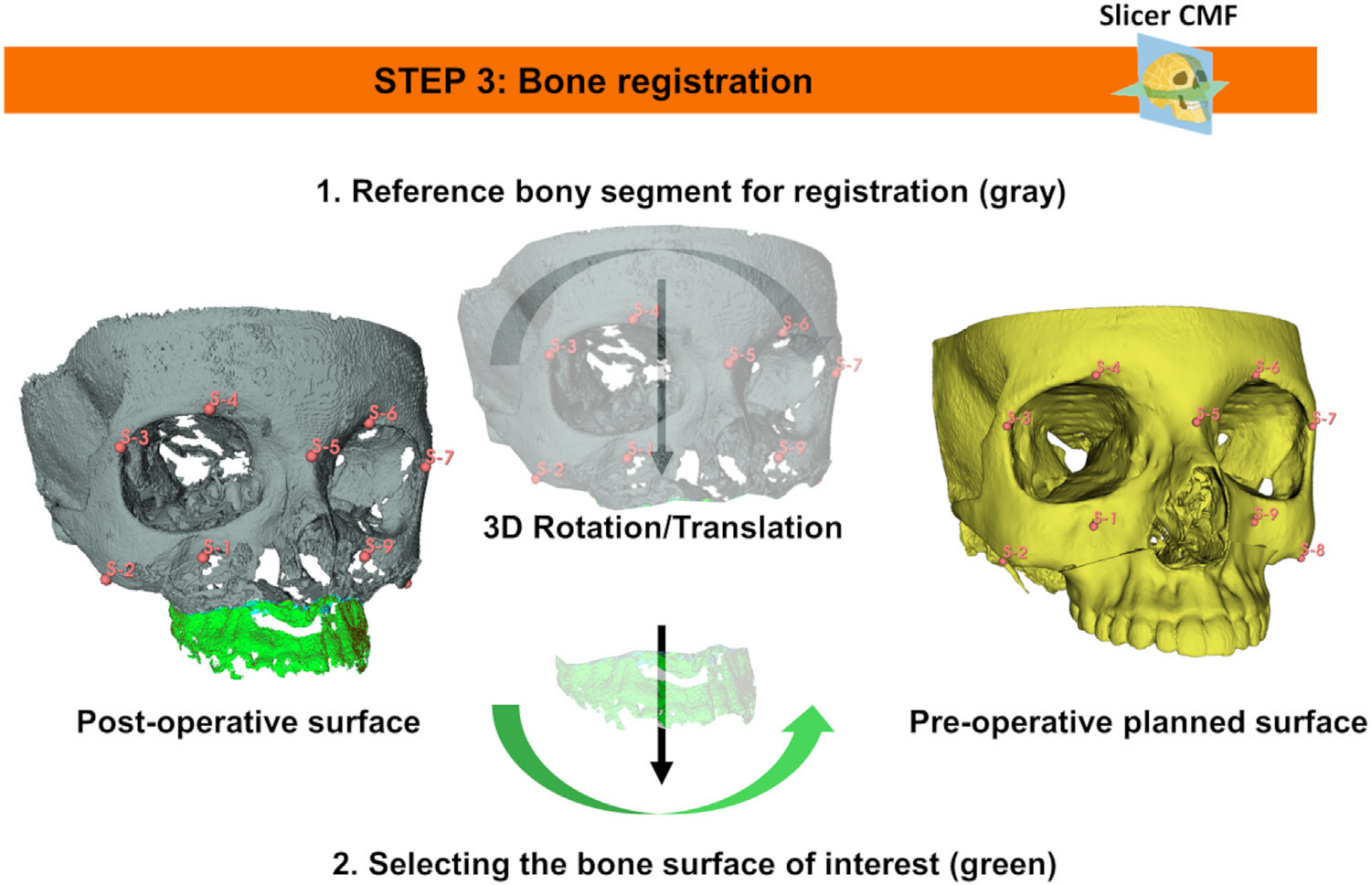
Post-operative surface models were registered onto pre-operative planning models using the *SlicerCMF* module in *3D Slicer*. Initially, landmark-based transformation (3D rotation and translation) aligned the post-operative model with the reference bony segment. Manual adjustment refined the alignment for optimal superimposition, and the final transformation was applied to the target bony segment.

**Figure 6:**
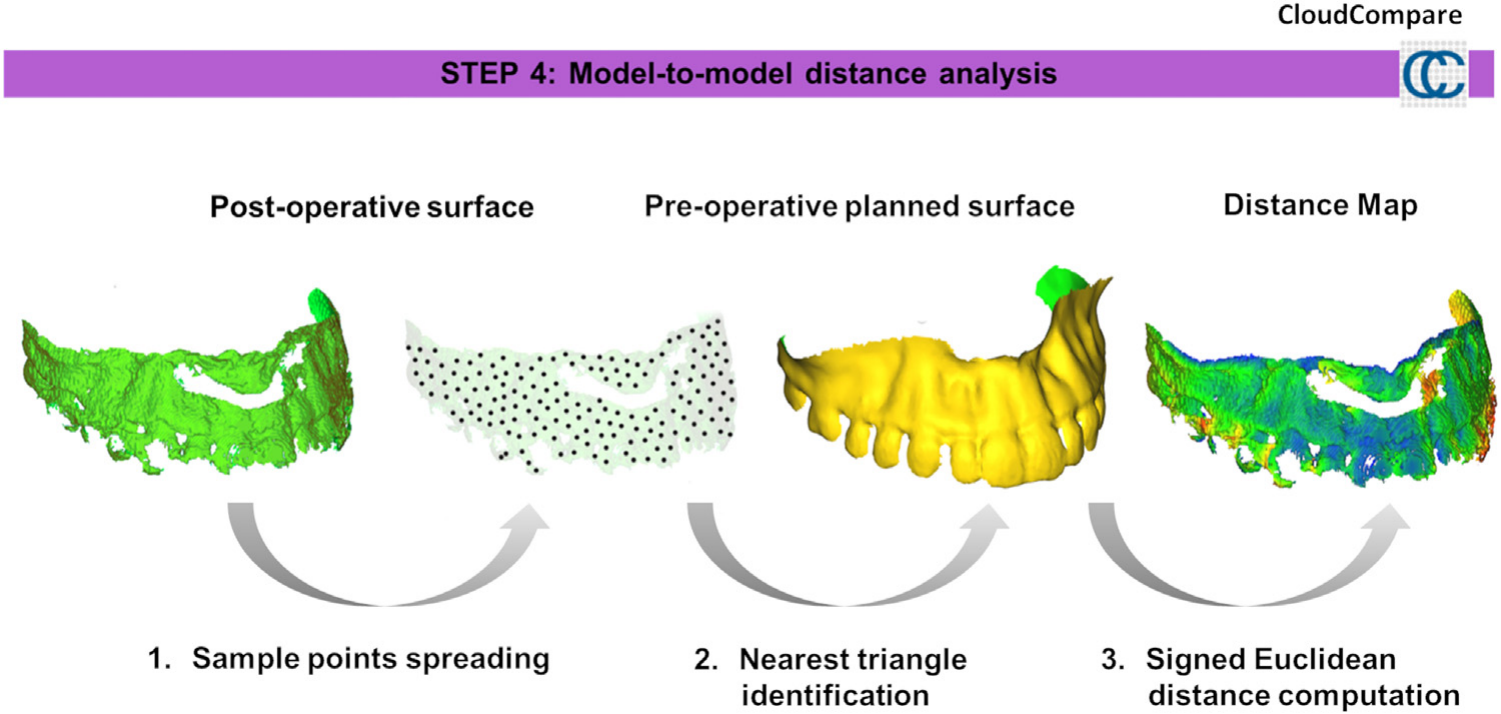
Distance between post-operative and pre-operative (reference) surface models was calculated using *CloudCompare* software. One million sample points were randomly distributed on the post-operative model, with the nearest triangle on the preoperative model identified for each. Signed Euclidean distances quantified surgical accuracy relative to the pre-operative planning.

**Figure 7:**
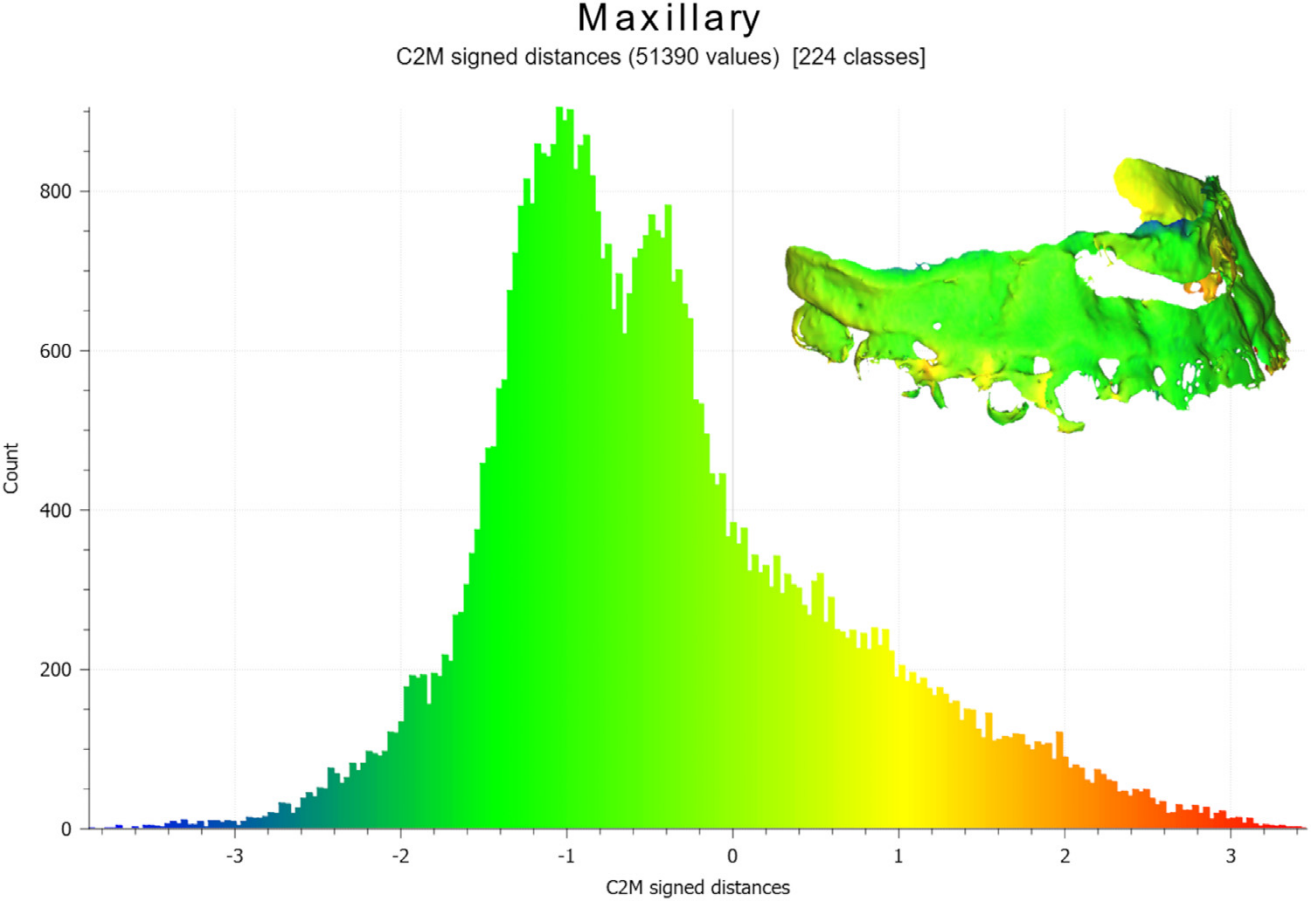
Graphics showing the distribution of values between post-operative and pre-operative maxillary surface models.

**Figure 8:**
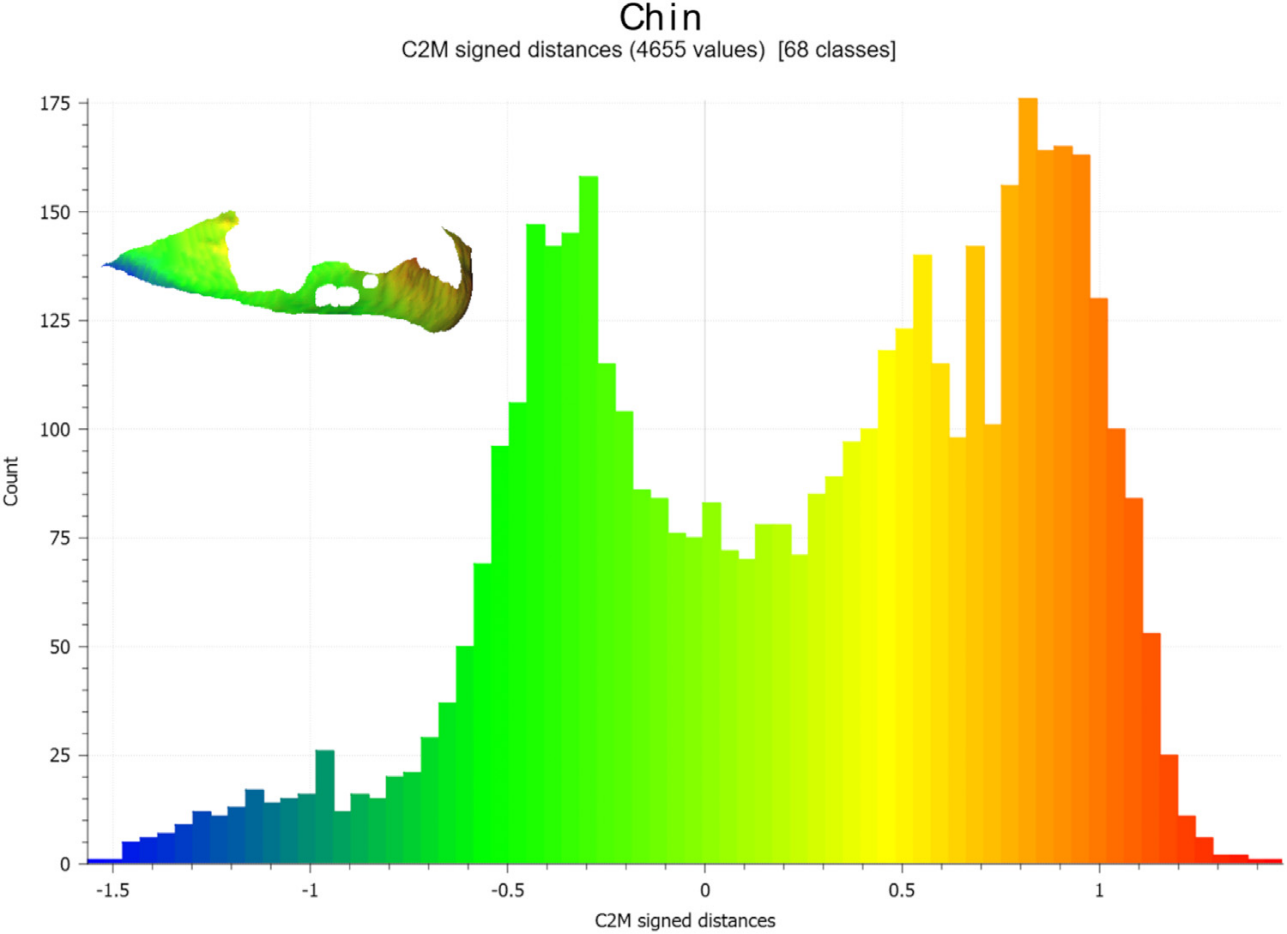
Graphics showing the distribution of values between post-operative and pre-operative chin surface models.

The CBCT scan most after the completion of orthodontic treatment was compared to the pre-operative planning scan to assess accuracy outcomes. This assessment followed the following procedure (Figure 3, Figure 4, Figure 5, Figure 6, Figure 7, Figure 8).

*Step 1: Bone Segmentation of Post-operative CT Images*

Post-operative bone segmentation was performed using the *Segment Editor* module of 3D Slicer software (version 5.6.1, https://www.slicer.org/).^31^ The *Otsu thresholding algorithm* was applied iteratively to determine an optimal threshold, minimizing intra-class variance between foreground and background pixels.^32^ This process produced isolated postoperative models of the skull, maxilla, mandible, and chin.

*Step 2: Extraction of Specific Surfaces*

The anterior surfaces of the maxilla and chin were extracted from their respective models. This was achieved using *Meshmixer* software (version 3.5.474, Autodesk Inc., USA) by manually selecting the surface of interest and exporting it as a new model. The same process was applied to the pre-operative planning models of the maxilla and chin, enabling precise model-to-model distance analysis.

*Step 3: Registration of Surface Models*

Post-operative surface models were registered onto pre-operative planning models using the following steps:

*Initial Registration*: The rigid transformation (3D rotation and translation) aligning the post-operative surface model with the pre-operative planning model of a reference bony segment was computed using landmarks registration via the *SlicerCMF*.^33^

*Manual Refinement****:*** The transformation was then manually adjusted to maximize model superimposition.

*Application of Transformation****:*** The resulting transformation was applied to the specific bony segment of interest. *A) Maxilla:* The skull was used as the reference bony segment, and nine anatomical landmarks were manually placed on both pre-operative and post-operative surface models: zygomatic angles, supraorbital notches, infraorbital foramina, fronto-zygomatic suture, and the nasion. *B) Chin:* The mandible served as the reference bony segment, with eight anatomical landmarks manually placed: bilateral interdental spaces between lateral incisors and canines, between first and second premolars, between first and second molars, and bilateral mental foramina. No landmarks were placed on the vertical branches of the mandible to avoid inaccuracies resulting from differential surgical modifications of the mandibular body and ramus.

*Step 4: Distance Computation*

The distance between the post-operative and pre-operative (reference) surface models was calculated using *CloudCompare* software (version 2.13.0, http://www.cloudcompare.org/). *Point Sampling:* One million sample points were randomly distributed across the post-operative surface model to ensure uniformity in the analyses. *Distance Calculation:* For each sample point on the post-operative surface model, the nearest triangle on the pre-operative planning model (reference) was identified. The *signed Euclidean distance* was computed for each sample point to quantify the accuracy of the surgical procedure relative to the pre-operative planning.

### Statistical analysis

Data were analyzed using *Matlab* (R2022b, The MathWorks, USA) and R statistical software (version 4.3.3; R Development Core Team, Vienna, Austria, 2024). The distribution of *signed Euclidean distances* between the post-operative surface model and the pre-operative planning surface model was visualized using boxplots for each patient.

The overall accuracy was classified into four categories based on the proportion of distances falling within the interval [−2 mm; +2 mm]:

• *Excellent:* >90 % of values within the interval.
• *Good****:*** 80 %–90 % of values within the interval.
• *Moderate****:*** 50 %–80 % of values within the interval.
• *Poor****:*** <50 % of values within the interval.

The [−2 mm; +2 mm] threshold range was established in a previous study and adopted here for consistency and comparability. The variability (standard deviation) of differences was also used as an indicator of accuracy. In addition, bivariate analyses assessed how overall accuracy (proportion of values within [–2 mm; +2 mm]) and variability (standard deviation) related to each predictor (gender, age, planned movements, skeletal class malocclusion). Mann–Whitney U or Kruskal–Wallis tests were used for categorical predictors, and Spearman’s rank correlation for continuous predictors. Significance was set at 0.05.

For cases with moderate or poor accuracy, the average primary direction of the positioning error was also qualitatively assessed and reported.

## Results

This study included 21 consecutive patients—19 Caucasians, 1 Asian, and 1 African—with a mean age of 19.1 years (range: 16–41). Of these, 11 (52.4 %) were men. All patients presented with non-syndromic facial asymmetry: 12 had skeletal Class II (5 of whom also had vertical maxillary excess) and 9 had skeletal Class III. Each patient underwent bimaxillary surgery, and 12 also underwent a genioplasty. Patient’s characteristics are summarized in Table 1.

**Table 1:** Patients’ characteristics.

Patient no	Gender	Age (years)	Etiology of asymmetry	Skeletal class malocclusion	Surgical planning
1.	M	19	Condylar fracture		Bimaxillary surgery
2.	F	41	Developmental	Class III	Bimaxillary surgery and genioplasty
3.	F	18	Developmental	Class III	Bimaxillary surgery and genioplasty
4.	M	16	Developmental	Class II and VME	Bimaxillary surgery and genioplasty
5.	F	19	Developmental	Class I	Bimaxillary surgery
6.	M	17	Developmental	Class I	Bimaxillary surgery and genioplasty
7.	F	20	Developmental	Class III	Bimaxillary surgery
8.	F	19	Developmental	Class III	Bimaxillary surgery
9.	M	17	Developmental	Class II and VME	Bimaxillary surgery and genioplasty
10.	F	17	Developmental		Bimaxillary surgery
11.	M	19	Developmental	Class III	Bimaxillary surgery and genioplasty
12.	M	18	Congenital muscular torticollis		Bimaxillary surgery
13.	M	17	Developmental	Class II and VME	Bimaxillary surgery and genioplasty
14.	M	19	Developmental	Class III	Bimaxillary surgery and genioplasty
15.	M	19	Developmental	Class III	Bimaxillary surgery
16.	M	18	Developmental	Class III	Bimaxillary surgery
17.	M	16	Developmental	Class I	Bimaxillary surgery and genioplasty
18.	F	19	Developmental	Class III	Bimaxillary surgery
19.	M	17	Developmental	Class II and VME	Bimaxillary surgery and genioplasty
20.	F	18	Juvenile rheumatoid arthritis	Class II	Bimaxillary surgery and genioplasty
21.	F	19	Developmental	Class II and VME	Bimaxillary surgery and genioplasty

Abbreviations: VME, vertical maxillary excess.

**Figure 9:**
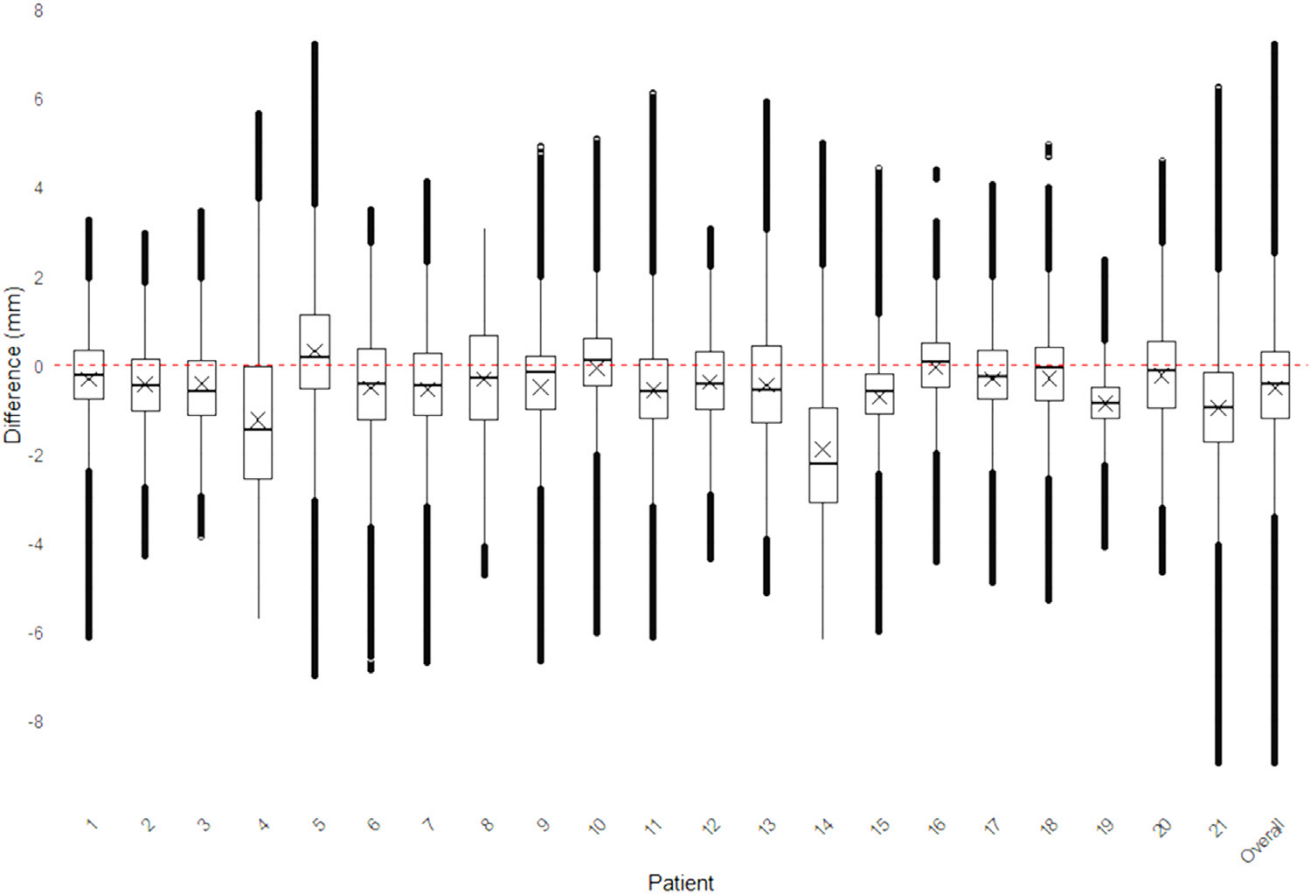
Boxplots of differences between the planned and final results in the 21 patients who underwent maxillary surgery. Mean values (X) are displayed.

**Figure 10:**
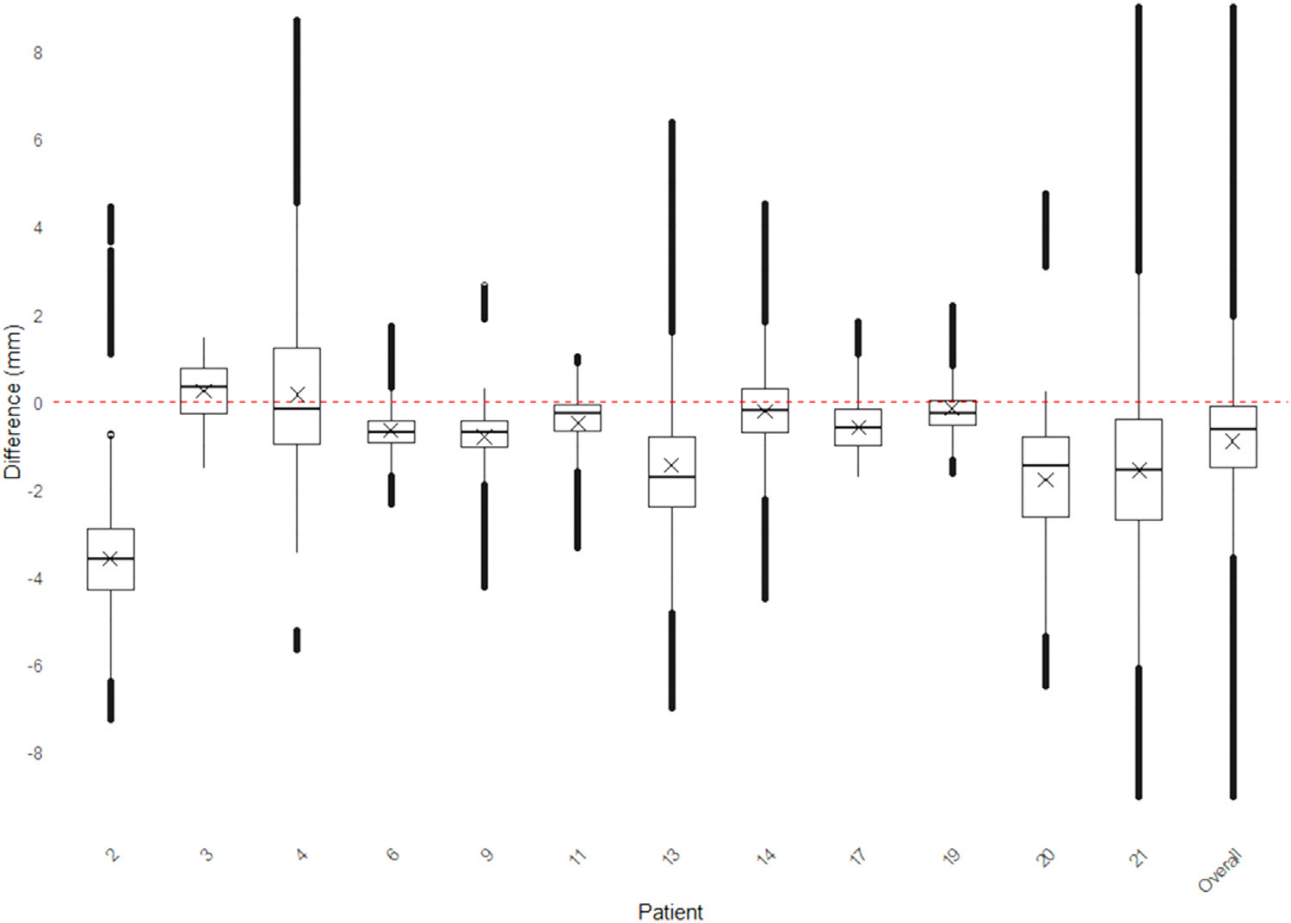
Boxplots of differences between the planned and final results in the 12 patients who underwent chin surgery. Mean values (X) are displayed.

Although the mean difference (indicated by X in Figure 9, Figure 10) between the planned and actual post-operative models was near zero for most patient, the overall variability was often substantial, with values spanning from −8 mm to +8 mm. Across all patients, the mean difference for maxillary and chin repositioning was very low at −0.9 mm and −0.5 mm respectively (Figure 9, Figure 10, last boxplot).

The overall accuracy for maxillary repositioning was assessed *Excellent* in 13 patients (62 %), *Good* in 5 patients (23.8 %), *Moderate* in 2 patients (9.5 %), and *Poor* in 1 patient (4.8 %).

The overall accuracy for chin repositioning was assessed *Excellent* in 7 patients (58.4 %), *Good* in none of the patients (0 %), *Moderate* in 4 patients (33.3 %), and *Poor* in 1 patient (8.3 %). The overall accuracy and variability were not significantly different according to patient characteristics (age, gender) or planned movements and skeletal class malocclusion.

All patients were informally asked at the end of treatment whether they were satisfied with the aesthetic outcome, and all expressed a positive response.

## Discussion

The aim of this retrospective study was to evaluate the 3D accuracy of maxillary and chin repositioning in non-syndromic patients with facial asymmetry using patient-specific implants (PSIs). Analysis based on mean differences between planned and post-operative models suggested that maxillary and chin repositioning could be accurately approximated and effectively predicted using PSIs.

Based on the assumption, supported by several researchers, that a 2 mm threshold for maxillary translational accuracy is clinically acceptable - given that differences within this range are unlikely to be perceptible to the naked eye or to patients - these findings further strengthen the evidence supporting the precision and efficacy of PSIs in orthognathic surgery, particularly for the treatment of facial asymmetry.^34^^,^^35^

To date, and to the best of our knowledge, no study has specifically focused on this distinct type of dento-skeletal deformity. Although some investigations have included patients with facial asymmetry, these cases were typically analyzed as part of broader study populations and not as a dedicated subgroup,^20^^,^^22^^,^^25^^,^^29^^,^^34^ Liu et al.^20^ remain the only authors to report on the use of PSIs in cases of facial asymmetry, with a specific focus on hemifacial microsomia. Their study compared the accuracy of CAD-CAM-fabricated wafers and PSIs in performing Le Fort I (LF1) repositioning. Accuracy verification was conducted by selecting three anatomical landmarks as reference points to measure differences in orientation between the planned and post-operative models. The PSI group demonstrated superior accuracy in LF1 spatial repositioning, with maximal deviations of <1 mm in translational and 1° in rotational movements. These findings highlight the significantly greater precision of PSIs in achieving planned surgical outcomes.^20^ However, it is important to acknowledge that their study was limited to a specific syndromic population, and accuracy was assessed using mean differences in distances and rotations between pre-operative and post-operative images—a method that may lead to an overly optimistic evaluation, as positive and negative discrepancies can cancel each other out.

A recent meta-analysis of 566 cases confirmed PSIs enhance orthognathic surgery accuracy, showing significantly less deviation than CAD-CAM splints and maintaining one-piece LF1 repositioning within clinically accepted thresholds (−2 mm, 4°).^25^

Consistent with previous studies in non-asymmetrical cases, our results demonstrate greater accuracy in maxillary repositioning compared to chin repositioning. We hypothesize that the smaller anatomical size of the chin and its exposure to muscular forces contribute to subtle deviations during surgery or healing. Additionally, some authors suggest that pre-operative mandibular positioning during CT imaging and concurrent orthodontic treatment may affect surgical precision. Variability in mandibular positioning or orthodontic forces may compromise chin stability. Recent studies further highlight the pivotal role of the chin in facial asymmetry correction.^36^, ^37^, ^38^, ^39^, ^40^ As a key determinant of facial balance, targeted interventions—particularly genioplasty and patient-specific implants—have proven effective in enhancing aesthetic outcomes, even in the absence of condylar surgery. These findings emphasize the importance of incorporating chin-focused strategies into personalized treatment plans for non-syndromic facial asymmetry.^36^, ^37^, ^38^, ^39^, ^40^

In cases with moderate or low accuracy, we observed a tendency to undercorrect, particularly in maxillary and chin advancement, resulting in slightly posteroinferior postoperative positions.^13^^,^^14^^,^^16^ As previously reported, this tendency may be partially due to bony interference and soft tissue retraction forces. These effects appear to be more pronounced in cases requiring larger movements, underscoring the importance of careful preoperative planning and intraoperative management to minimize displacement and optimize outcomes.^15^^,^^20^^,^^21^

Although PSIs provide high accuracy for maxillary and chin repositioning, we chose not to use them for mandibular osteotomies for several reasons. First, as highlighted by Heufelder et al.^14^ we lacked confidence in virtual condyle positioning and preferred manual control to ensure optimal placement. This is consistent with the concerns raised by Badiali et al.,^22^ who emphasized that precise control of virtual condyle positioning is critical, as overly conservative ramus placement may increase interference between mandibular fragments.

No studies have yet demonstrated significant advantages of PSIs for mandibular repositioning. Shakoori et al.^24^ compared PSI-based maxillary and mandibular osteotomies and reported significantly greater variability in mandibular landmarks and even cautioned against PSI use in cases of pronounced mandibular asymmetry.

Several authors have documented complications associated with mandibular PSIs. Some authors reported fitting problems in more than half of their cases, while others recommended fabricating splints as a backup in case PSIs do not fit properly.^12^^,^^22^ Given these challenges, we opted for a more controlled approach to mandibular repositioning to minimize potential inaccuracies and complications.

The primary strength of this study is its uniqueness as the first series to evaluate the accuracy of maxillary and chin repositioning in patients with non-syndromic facial asymmetry using PSIs. Data were collected during multidisciplinary clinical examinations, with clinicians blinded to the purpose of the study at the time of evaluation to ensure unbiased observations.

Box plots were used to analyze differences between planned and final outcomes, highlighting outliers. Assessments by experts improved reliability, and inclusion of all eligible patients minimized selection bias, ensuring a comprehensive and representative dataset for the study.

However, the findings of this study should be interpreted with consideration of certain limitations: (1) its retrospective design within a single institution, which may limit generalizability; (2) the inability to fully control for potential biases or errors associated with both the computer-assisted 3D virtual planning and PSI design, as well as the accuracy assessment procedure itself; (3) the relatively small patient cohort, which may affect the statistical power of the analysis; and (4) the absence of a control group consisting of patients who underwent the same procedure using splints, preventing direct comparative evaluation of PSI-based and conventional approaches.

## Conclusion

This study demonstrated that planned surgical outcomes for maxillary and chin repositioning in non-syndromic patients with facial asymmetry can be accurately approximated using PSIs, with greater accuracy observed in maxillary repositioning compared to the chin.

Larger studies with expanded datasets should explore additional factors such as surgeon experience, implant design, and soft tissue behavior to further improve the accuracy and predictability of PSIs in managing complex dento-skeletal deformities.

## Ethical approval

The study was conducted in accordance with the Declaration of Helsinki and was approved by our local Ethical Board (Project ID 2018–00,754).

## Declaration of competing interest

None.

## Appendix Supplementary materials

Image, application 1
